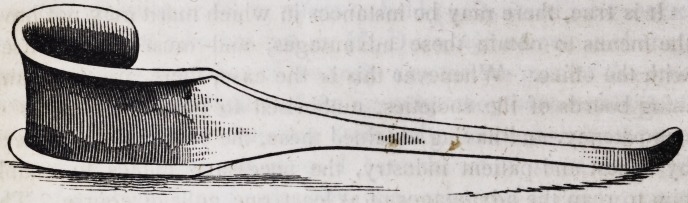# The Cheek and Lip Protector, (or Retractor.)

**Published:** 1844-03

**Authors:** J. S. Gunnell


### ARTICLE VI.

### *The Cheek and Lip Protector, (or Retractor.)*

By J. S. Gunnell, M. D.

The protector, for opening the mouth and drawing the cheek and lips backwards, protecting them from injury, &c. (invented and used by me ever since the year 1823,) is a silver instrument, (though any other precious metal will answer,) about six or eight inches long, with a handle or stem, and a curve at one or both ends, the curved part about one inch wide. When the curved part is put in the mouth, the inner part of the curve is to be next to the cheek, and the hollow or flexed, or outer part of the curve, next to the front, or forward, and the handle pointing towards or beyond the ear, and backwards; by the instrument being thus applied, the lips and cheeks may be freely drawn backwards, or to one side, and thereby bring the whole of that side of the mouth in view of the operator, which will enable him to file any of the back teeth, or perform any other operation on the teeth, without difficulty, and the lips and cheek be guarded from injury from the file, by the file passing in the hollow on the outer or back part of the protector, and between the curved edges or flanges of the instrument. The protector serves all other necessary purposes of opening the mouth and protecting the soft parts from injury during the operation of the dentist.

## Figures and Tables

**Figure f1:**